# Trends in Obesity among Iranian Children and Adolescents: 2000–2016

**Published:** 2020-01

**Authors:** Ensiyeh Jenabi, Salman Khazaei

**Affiliations:** 1 Autism Spectrum Disorders Research Center, Hamadan University of Medical Sciences, Hamadan, Iran. 65178-38678. Tel: +98 8138380717. Fax: +98 8138380130. Email: en.jenabi@yahoo.com.; 2 Assistant Professor of Epidemiology, Research Center for Health Sciences, Hamadan University of Medical Sciences, Fahmideh Street, Hamadan, Iran. 65178-38678. Tel: +98 8138380717. Fax: +98 8138380130. Email: salman.khazaei61@gmail.com.

Dear Editor in Chief,

Childhood obesity is an important predisposing factor for most noncommunicable diseases, namely diabetes, high blood pressure, asthma and other respiratory problems, sleep disorders, and liver disease in the future.^1^ Environmental and cultural factors, as well as lifestyle preferences, play important roles in the rising prevalence of childhood obesity worldwide.^2^ Evidence implies the increasing trend of childhood obesity globally, especially in developing countries.^3^^, ^^4^ Studies in developing countries are more concentrated on nutritional deficiencies. Deficiencies in nutrition, notwithstanding, childhood obesity and its complications should be focused upon for appropriate practice of evidence-based health promotion.^5^ Accordingly, we sought to collect evidence on the prevalence and trends of obesity among children and adolescents in Iran and compare our findings with the other regions of the World Health Organization (WHO) using the WHO data. ^6^

As is shown in Figure 1, the trend of obesity for both boys and girls in the age brackets of 5 to 9 years and 10 to 19 years in Iran had an increase between the years 2000 and 2016. In both age groups, the prevalence of obesity in all years was higher in boys. For boys aged between 5 and 9 years, the prevalence of obesity reached 13.1% in 2016 from 6% in 2000 (P for trend <0.05); while for girls between 5 and 9 years of age, the figure reached 9.8% in 2016 from 4.9% in 2000 (P for trend <0.05). Concerning adolescents, the obesity prevalence for boys between 10 and 19 years old reached 9.3% in 2016 from 3.9% in 2000; whereas for girls in the same age bracket, the figure reached 8.1% in 2016 from 3.9% in 2000 (P for trend <0.05).

Figure 2 depicts a comparison in terms of the prevalence of obesity between children and adolescents in Iran and those in the other WHO regions in 2016. In this year, 18% of children and adolescents aged between 5 and 19 years were overweight or obese. The highest prevalence of obesity among both children and adolescents, be they boys or girls, was in the Americas (14% and 11.5% for boys and girls aged 10–19 years, respectively, and 19.3% and 15% for boys and girls aged 5–9 years, correspondingly). Also in the year 2016, South East Asian countries had the lowest rate of the mentioned indicators (2.7% and 1.8% for boys and girls aged 10–19 years, correspondingly, and 5.2% and 3.6% for boys and girls aged 5–9 years, respectively). The prevalence rate of obesity for boys between 5 and 9 years of age in Africa was only 2.8%. The prevalence rate of obesity for the aforementioned indicators in Iran was higher than that in South East Asia, Africa, Europe, which was much higher than the global average (for boys aged 10–19: 9.3% vs. 6.5% and for girls aged 10–19: 8.1% vs. 4.7%; for boys aged 5–9 years: 13.1% vs. 10.4% and for girls aged 5–9: 9.8 vs. 7.5%).

**Figure 1:**
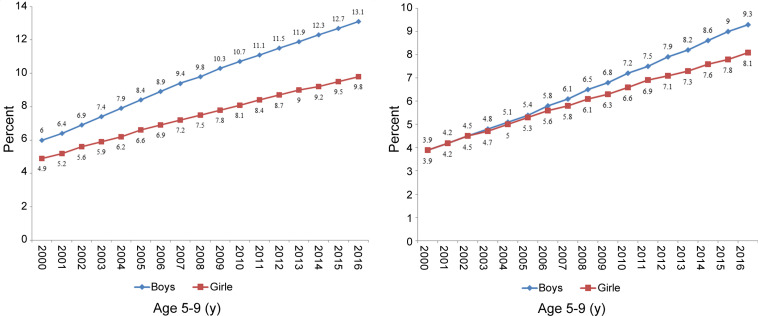
Trend in the prevalence of obesity among Iranian children and adolescents (2000–2016)

**Figure 2:**
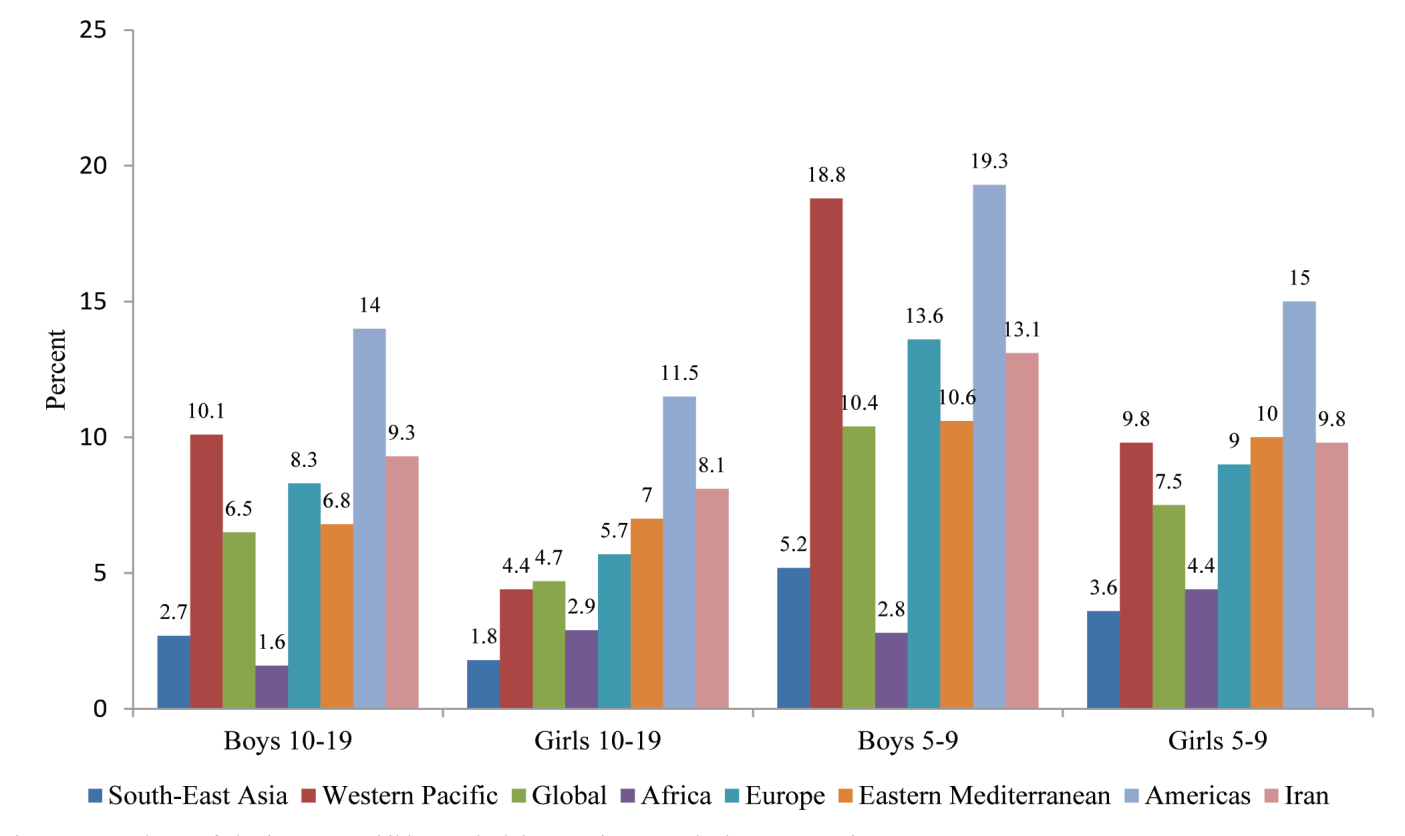
Prevalence of obesity among children and adolescents in Iran and other WHO regions

## Conclusion

The high prevalence and increasing trend of obesity among children and adolescents must be deemed alarming by health policymakers, who should devise interventional preventive programs aimed at these sensitive age groups. The high prevalence of obesity in adults in many countries renders weight reduction a tremendous challenge; it is, therefore, advisable that children be considered the priority population for such intervention strategies as enhanced physical activity and improved diet. Preschool institutions and schools are appropriate places for the implementation of these interventions.
